# Prenatal diagnosis of intrathoracic kidney and adrenal gland in left-sided congenital diaphragmatic hernia

**DOI:** 10.1007/s00404-024-07449-7

**Published:** 2024-03-09

**Authors:** Tian Tian, Huizhu Chen, Jing Tang, Hong Luo

**Affiliations:** 1 Department of Ultrasound, West China Second University Hospital, Sichuan University, Chengdu, 610041 Sichuan China; 2 Department of Radiology, West China Second University Hospital, Sichuan University, Chengdu, 610041 Sichuan China; 3 Department of Radiology, West China Hospital of Sichuan University, Chengdu, 610041 Sichuan China; 4 Key Laboratory of Birth Defects and Related Diseases of Women and Children (Sichuan University), Ministry of Education, Chengdu, 610041 Sichuan China

**Keywords:** Intrathoracic kidney, Ectopic adrenal gland, Bochdalek hernia, Prenatal diagnosis

## Abstract

A 29-year-old primigravida at 31 weeks of gestation was referred for intrathoracic kidney (ITK). Ultrasound revealed left kidney intrathoracic placement with an anteriorly positioned ectopic adrenal gland. Magnetic resonance imaging confirmed diaphragmatic interruption and colon herniation. A female neonate, delivered at 37 weeks, underwent successful thoracoscopic repair for a left Bochdalek hernia. Despite compression of the left lung, notably optimistic lung-to-head ratio (LHR) values were observed, correlating with favorable outcomes. This case underscores the rare occurrence of ITK, its association with Bochdalek hernia, and the importance of comprehensive prenatal evaluations.

## What does this study add to the clinical work

**Table:** 

Detailed prenatal imaging evaluation facilitates better planning of neonatal period management, and ITK in combination with congenital diaphragmatic hernia tends to exhibit a favorable prognosis.	

## Presentation

**Fig. 1:**
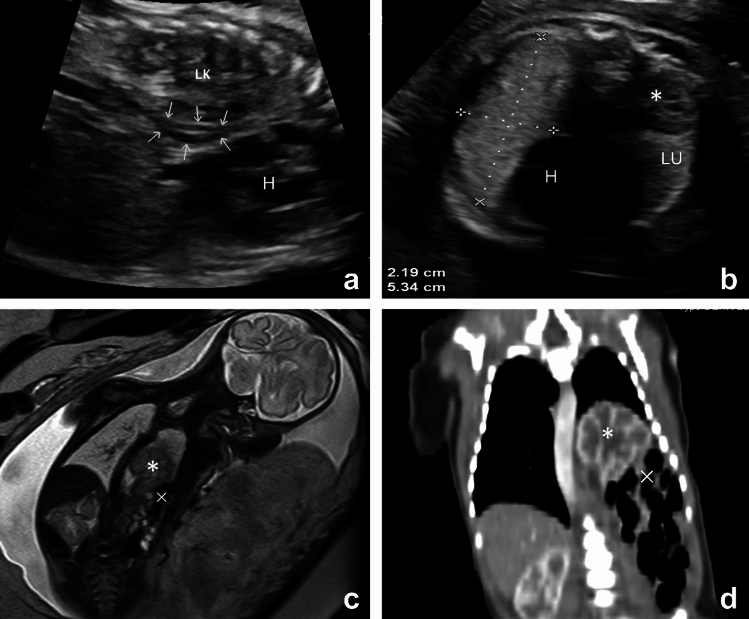
Prenatal and postnatal image of intrathoracic kidney (ITK). **a** Sagittal ultrasound image of the left fetal thorax at 31 weeks, revealing the left kidney located in the posterior-inferior left thoracic cavity, and adrenal gland positioned between the ITK and the heart (LK: left kidney, arrow: left adrenal gland, H: heart). **b** Transverse ultrasound image of the fetal thorax at 31 weeks, illustrating the measurement of the lung-to-head ratio (LHR). (*: left kidney, H: heart, LU: left lung).** c** Coronal T2-weighted MRI images at 32 weeks’ gestation, displaying herniation of the left kidney and a small portion of the colon (*: left kidney, × : colon). **d** Postnatal enhanced CT on day 1 of life (*: left kidney, × : colon)

A 29-year-old primigravida at 31 weeks of gestation was referred for intrathoracic kidney (ITK). Ultrasound imaging disclosed the left kidney’s intrathoracic location, accompanied by an anteriorly positioned ectopic adrenal gland (Fig. 1a). The ITK exhibited no appreciable alterations in size or echogenicity. Notably, color Doppler imaging revealed the left renal artery’s origin from the abdominal aorta, extending cranially into the ITK. Left lung compression was observed, with a lung-to-head ratio (LHR) of 4.2 and an observed-to-expected (O/E) LHR of 98.4% (Fig. 1b). Fetal echocardiogram and other structural screening revealed no abnormalities. Magnetic resonance imaging (MRI) confirmed diaphragmatic interruption, with herniation of a small colon portion through the defect (Fig. 1c). Genetic amniocentesis was declined. A female neonate, weighing 2,500 g, was delivered at 37 week’s gestation, with Apgar scores of 10 at 1 and 10 min. Postnatal enhanced CT findings (Fig. 1d) aligned with prenatal MRI. Thoracoscopy on day 4 confirmed a left Bochdalek hernia (7 cm × 7 cm × 8 cm) with a hernial sac and a diaphragmatic defect (3 cm × 5 cm). Successful primary closure was performed, and the infant had an uneventful hospital course, currently thriving at 42 months.

## Discussion

Intrathoracic kidney is exceedingly rare, with limited prenatal diagnoses [1], and scant mention of adrenal gland location in such cases [2]. Ipsilateral adrenal glands often display a “lying-down” sign in pelvic ectopic kidneys [3], while in ITK, they may reside in situ or the thorax [4]. Prenatal MRI, revealing both diaphragmatic defect and colon herniation, clarified the diagnosis despite the initial consideration of a diaphragm-intact ITK during ultrasound. ITK frequently coexists with Bochdalek hernia, characterized by a defect in the posterolateral aspect of the diaphragm. Neonates affected by this conjunction typically exhibit a favorable prognosis, corroborating with established observations [1, 5]. Nonetheless, our study underscores the imperative of comprehensive prenatal imaging assessments for ITK.

## Data Availability

The data presented in this study are available in article.
